# Obesity and Midlife Performance: A Cross‐Sectional and Longitudinal Analysis of Women in the Menarche‐To‐PreMenopause Study

**DOI:** 10.1111/dom.71349

**Published:** 2026-09-14

**Authors:** Leticia W. Ribeiro, Jenny Doust, Gregore I. Mielke, Gita D. Mishra

**Affiliations:** ^1^ Australian Women and Girls' Health Research Centre, School of Public Health The University of Queensland Brisbane Queensland Australia

**Keywords:** anthropometry, handgrip strength, obesity, physical performance, women's health

## Abstract

**Background:**

The interplay between obesity and physical performance in women is poorly understood, especially before the fifties.

**Objective:**

To assess associations between weight gain from early to mid‐adulthood and midlife performance‐based physical function.

**Methods:**

499 women from the Menarche‐to‐PreMenopause Study completed handgrip strength, chair rise, and standing balance tests at age 41–48. The lowest tertile was defined as the ‘worse performance’ group. Weight and height were self‐reported across eight surveys, from ages 18–23 to 40–45, and used to determine body mass index (BMI) trajectories using group‐based trajectory modelling, as well as to estimate annual weight gain percentages. Other obesity indicators included BMI, waist circumference (WC), and waist‐to‐hip ratio (WHR) measured cross‐sectionally at age 41–48.

**Results:**

Two BMI trajectories were identified: Normal‐Increasing (75%) and High‐Increasing (25%). The High‐Increasing BMI trajectory showed approximately 40% higher prevalence of worse chair‐rise and standing‐balance performances compared to the Normal‐Increasing group (95% CI: 9%–84%, *p* = 0.01; and 10%–84%, *p* = 0.008). Higher annual weight gain percentage showed a prevalence ratio (PR) 1.21 times higher for worse standing balance performance (95% CI: 1.06–1.38, *p* = 0.004). Midlife obesity (BMI ≥ 30 kg/m^2^), high‐risk WC (≥ 80 cm) and WHR (≥ 0.8) were also associated with a higher prevalence of worse performance on the chair rise and standing balance tests, with PRs from 1.41 to 1.55. No consistent evidence was found for handgrip strength.

**Conclusions:**

Weight gain from early to mid‐adulthood was associated with worse performance on the chair rise and standing balance tests in midlife, highlighting the potential of early weight‐control strategies to preserve midlife performance.

## 1 Introduction

Overweight and obesity are an increasing public health concern, with significant detrimental health consequences, such as the development of Type 2 diabetes, cardiovascular disease, some types of cancer, mental health disorders, and other life‐course problems [1, 2]. Global projections for 2050 indicate that if current trends continue, the prevalence of overweight and obesity will affect more than half of the adult population worldwide [2]. As of 2022, 66% of Australian adults were already living with overweight or obesity, following a steady, substantial increase over the past three decades, from 20% in 1995 [3]. The proportion of Australian women with obesity was ranked ninth across 21 member countries of the Organisation for Economic Co‐Operation and Development (30%) [4].

Marked weight gains occur from women's young adulthood onwards, with significant interactions with life milestones such as marriage and childbearing [5, 6]. From the eighties, BMI starts to decline due to lean mass and fat mass loss, increasing the risk of frailty and disability [6]. Nonetheless, the prevalence of hidden conditions such as sarcopenic obesity has been growing in ageing populations with the obesity epidemic [7]. Sarcopenia is a key component of frailty, characterised by loss of muscle function (particularly muscle strength) and mass, which might be masked by excessive fat mass in those with obesity. Studies suggest that weight and weight change from earlier life stages may be potential risk factors for frailty at older ages [6, 7, 8].

It is important to acknowledge that being underweight also impacts health. A recent systematic review and meta‐analysis showed a pooled OR for frailty of 1.70 (95% CI: 1.13, 2.57) associated with being underweight and OR 1.70 (95% CI: 1.21, 2.51) associated with obesity [9]. However, only two of the seven cohort studies included in this study looked at BMI in middle age and the association with frailty at older age. Moreover, sex differences were not thoroughly discussed in this review. The review found positive associations between fat mass and frailty indexes in women, but not in men. Our previous systematic review of life course factors associated with handgrip strength, one of the diagnostic criteria for sarcopenia, showed inconclusive results among 12 studies examining BMI, with some indicating male‐specific associations [10]. Thus, this study aims to evaluate the associations between weight indicators from ages 18–23 to 40–45 and performance‐based physical function tests at age 41–48 among mid‐aged Australian women.

## 2 Materials and Methods

This study used data from the Menarche‐to‐PreMenopause (M‐PreM) Study, a sub‐study of the Australian Longitudinal Study on Women's Health (ALSWH) that investigated associations between reproductive factors and the risk of chronic diseases. More information about the ALSWH, a national flagship study running since 1996, and the cross‐sectional M‐PreM Study can be found elsewhere [11, 12]. In summary, M‐PreM ran between 2019 and 2021, including a period of study suspension (March–November 2020) due to the COVID‐19 pandemic. Of the eligible women from the ALSWH cohort born in 1973–1978 (*n* = 7121), 4584 expressed interest and 1278 participated in the sub‐study. Those women were invited to attend a clinical assessment at one of the study clinics situated in Brisbane, Melbourne, Sydney, Adelaide, or Perth.

This study comprises 499 women who attended a single clinical assessment in M‐PreM, during which functional tests and anthropometric measurements were performed by trained local research nurses (Appendix 1, Supplementary Figure 1). One trained research nurse conducted each assessment in its entirety, with nurses varying across clinic sites.

Compared to those who completed a self‐assessment or did not participate in M‐PreM, clinic participants were more urban, more highly educated, better able to manage their income, and had fewer comorbidities. They were also more physically active, less likely to have ever smoked, and more likely to be low‐risk drinkers rather than never/rarely or high‐risk drinkers (Appendix 1, Supplementary Table 1).

M‐PreM was approved by the Metro South Hospital and Health Services Human Research Ethics Committee [HREC/2019/QMS/52052] and the Human Research Ethics Committees of the Universities of Newcastle and Queensland [2004/HE000224], and all participants provided informed consent.

### 2.1 Performance‐Based Physical Function Tests

Three physical function tests were administered by research nurses at M‐PreM clinical assessments, when participants were aged 41–48. Handgrip strength was measured in kilogrammes using three identical Jamar hydraulic hand dynamometers (model 503JI) [13]. The same accredited provider calibrated the devices before reuse at subsequent clinic sites. Participants were seated and asked to squeeze the handle of the dynamometer while holding their elbow against their side at a 90° angle with their forearm and wrist in a neutral position. The greatest value of two measurements (one in each hand) was used in the analysis.

The chair rise test measured the time in seconds (sec) to complete ten sets of a sit‐stand‐sit manoeuvre. Participants performed the test without shoes in a straight‐backed, armless chair with a horizontal, flat seat. The reciprocal time was used in the analysis, so that higher scores represented better performance, similar to the other tests [14].

Standing balance was measured as the maximum time (up to 30 s) holding a one‐legged stance, first with eyes open and then closed. Participants were asked to remove their shoes, cross both arms over the chest, and stand on their preferred foot while raising the other foot off the floor by bending their knee. Standing balance with eyes closed was used in the analysis to avoid a ceiling effect [14].

Tests were conducted in the following order: handgrip strength (left hand before the right), standing balance test (eyes open before closed), and chair rise test. No formal rest interval was prescribed between the tests. Performance in each physical function test was categorised according to the sample's tertiles. The lowest tertile was called the ‘worse performance’ group, and the two highest tertiles represented ‘better performance’. Results were missing for handgrip strength (*n* = 13), chair rise (*n* = 4), and standing balance (*n* = 1).

### 2.2 Weight Indicators

Cross‐sectional anthropometric measurements include weight, height, and waist and hip circumferences evaluated by research nurses during M‐PreM clinical assessments. Participants removed their shoes and were weighed to the nearest 0.1 kg using a digital scale, while height was measured to the nearest 0.1 cm using a stadiometer. We relied on the clinics' devices and calibration protocols for both measurements. A measuring tape was provided to each clinic to measure circumferences. Waist circumference (WC) was measured in centimetres at the middle point between the bottom of the last palpable rib and the top of the iliac crest. The hip circumference was measured at the most prominent point of the buttocks. Measurements were taken two times by the same research nurse, or three if there was a difference greater than 0.5 cm between the first two. The average circumferences were used in the analyses.

BMI was calculated by dividing weight in kilogrammes by the square of height in metres. Then, BMI was classified into ‘Under/Normal weight’ (BMI < 25 kg/m^2^; as only two participants were underweight, i.e., BMI < 18.5 kg/m^2^), ‘Overweight’ (≥ 25 kg/m^2^ and < 30 kg/m^2^) and ‘Obesity’ (≥ 30 kg/m^2^). WC served as an indicator of abdominal fat distribution, categorising a WC > 80 cm as high risk of chronic disease [15]. The waist‐to‐hip ratio (WHR) was calculated (WC/hip circumference) and categorised as high risk using a 0.8 cutoff [16]. BMI was missing for one participant, as was hip circumference.

Longitudinal anthropometric measurements include triennial self‐reported weight (in kg) and height (in cm) in ALSWH Surveys 1–8, i.e., from ages 18–23 to 40–45 (1996–2018). Self‐reported measurements showed strong agreement with measured height, weight and BMI in previous ALSWH data [17]. Missing weight and BMI values were present in Survey 1 (*n* = 36), Survey 2 (*n* = 97), Survey 3 (*n* = 94), Survey 4 (*n* = 43), Survey 5 (*n* = 52), Survey 6 (*n* = 27), Survey 7 (*n* = 25), and Survey 8 (*n* = 3). The median number of weight measurements per participant was 8 (7, 8).

Percent change in weight at each ALSWH Survey was calculated as the difference in weight between two consecutive surveys divided by the original weight in the first survey, multiplied by 100. Next, the annual percent change in weight was calculated using the compound annual growth rate formula to reflect the 3 years between surveys: [(1 + percent change/100)^(1/3)^ − 1] × 100. The average annual percent change in weight was calculated for participants with change data from at least two surveys (*n* = 491).

### 2.3 Covariates

Covariates were selected a priori based on the literature [14, 18, 19, 20, 21], including age at baseline and M‐PreM clinical assessment, and education, marital status, presence of comorbidities, respiratory conditions, smoking status, alcohol consumption, and physical activity from ALSWH Survey 8. Clinic location (Brisbane, Melbourne, Adelaide, Perth, Sydney) was included to account for potential site‐specific differences in physical function assessments and anthropometric measurements.

Menopausal status was considered as a potential covariate. Women were classified as pre‐, peri‐, or post‐menopausal according to self‐reported menstrual patterns. Women with hysterectomy (*n* = 6) or using hormonal treatments (*n* = 178) were classified as having unknown menopausal status. Post‐menopausal women included those with natural (*n* = 11) or surgical (*n* = 1) menopause [22].

Education was measured by the highest qualification achieved (‘Diploma or less’, ‘University degree’, ‘Higher university degree’). Some studies suggest that wealth might be more consistently associated with physical function than education [23], and therefore, the ability to manage income was also considered. Participants were asked, ‘How do you manage on the income you have available?’ and answers were grouped into ‘Impossible/Difficult’ (representing those who replied it is impossible, difficult all the time, or difficult some of the time), ‘Not too bad’, and ‘Easy’. Marital status was categorised into ‘Never married’, ‘Married/De facto’, and ‘Separated/Divorced/Widowed’. Self‐reported presence of comorbidities included osteoarthritis, rheumatoid arthritis or other arthritis (*n* = 52), diabetes (*n* = 13), heart disease (*n* = 4), and cancer (*n* = 74). Respiratory conditions, including asthma and bronchitis, were analysed separately [14].

Physical activity (PA) levels were categorised as ‘Nil/Low’, ‘Moderate’, and ‘High’ based on the total volume of activities during the previous week, including brisk walking, moderate and vigorous leisure activities, expressed in MET.minutes (0 to < 500; 500 to < 1000; and ≥ 1000 MET.minutes/week, respectively) [24]. As in ALSWH Survey 1 (ages 18–23), PA levels were evaluated using a different recreational physical activity score [25], Survey 2 was used as the baseline PA measure. Smoking status was coded as ‘Never smoker’ and ‘Ex/Current smoker’. Alcohol drinking pattern was classified into ‘Non‐drinker/Rarely drinks’, ‘Low risk’, and ‘Risky/High risk drinker’ according to recommendations from the National Health and Medical Research Council [26]. Values were missing for menopausal status (*n* = 1), presence of comorbidities (*n* = 1), and PA levels (*n* = 84 at Survey 2; and *n* = 14 at Survey 8).

### 2.4 Statistical Analysis

Continuous variables were presented as mean and standard deviation (SD) or median and interquartile range (IQR), while categorical variables were expressed as counts (*n*) and percentages (%). Chi‐square, ANOVA, and Kruskal–Wallis tests were used to investigate differences in characteristics between performance and three BMI groups at ages 41–48. Locally weighted scatterplot smoothing (LOWESS) was used to visually examine the cross‐sectional associations between performance and objective anthropometric measurements at ages 41–48 years.

Poisson regression models with robust error variance were used to examine the cross‐sectional relationships between weight indicators and worse performance (vs. better performance) on the physical function tests. Model 1 contained the analysis adjusted for clinic location, age, and physical activity, and Model 2 was further adjusted for education, income, marital status, presence of comorbidities, respiratory conditions, smoking status, and alcohol drinking pattern.

Group‐based trajectory modelling was conducted using the *traj* command in Stata to identify BMI trajectories over the 22 years of follow‐up, based on self‐reported measures from ALSWH surveys 1–8 [27]. Maximum likelihood estimation used all available BMI data points for each participant without needing to drop incomplete records or impute missing values. The model selection criteria included lower Akaike's Information Criterion (AIC) and Bayesian Information Criterion (BIC), and higher entropy values (entropy = 0.96). Additionally, a trajectory size of at least 5% of the sample and an average posterior probability of group membership of at least 0.7 were considered [28]. The selected trajectory model was characterised by a censored normal distribution and assumed a linear relationship with two distinct groups. Increasing the number of trajectory groups beyond two did not improve model fit based on BIC and produced small groups (< 5% of participants), indicating a less parsimonious solution.

Percentage weight change was calculated among participants with change data from at least two surveys to provide a continuous measure of relative change in body weight between surveys, complementing the trajectory‐based analysis. The BMI trajectory groups and the average annual percent change in weight were then added as exposure variables in separate Poisson regression models to evaluate respective associations with performance at ages 41–48. Model 1 was adjusted by clinic location, baseline age, physical activity levels at ALSWH Survey 2 (baseline) and M‐PreM/ALSWH Survey 8 (final follow‐up), and height (for annual percent change only). Model 2 included additional adjustment for education, income, marital status, comorbidities, smoking status and alcohol intake at final follow‐up. A directed acyclic graph (DAG) can be found in Appendix 2, Supplementary Figure 2.

All available data for each participant were included in the Poisson regression models. Those with incomplete covariate data (*n* = 95) were slightly younger, with a lower proportion of women finding it easy to manage their income, and a larger proportion of ex/current smokers. No other significant differences in characteristics were found (Appendix 2, Supplementary Table 2).

Sensitivity analyses assessed the robustness of findings by additionally adjusting for menopausal status, examining models adjusted for baseline or final follow‐up physical activity separately, and repeating analyses using multiply imputed covariate data. Multiple imputation generated 40 datasets using Stata's *mi impute* command with a fixed random‐number seed, and estimates were combined using *mi estimate* [29].

All analyses were performed using Stata (SE‐Standard edition, version 17.0; StataCorp, College Station, TX). An alpha level of *p* < 0.05 was considered statistically significant.

## 3 Results

Sample characteristics are shown in Table 1. There were equal proportions of women with overweight and obesity in the sample (39.5%). Women with obesity showed lower levels of physical activity, lower education, more difficulties managing income, with a higher proportion never having married and being ex/current smokers than women in the under/normal weight and overweight groups. Among the 334 participants (66.9%) with high risk of chronic conditions according to WC, 40% (*n* = 134) were overweight and 44% (*n* = 147) had obesity. A total of 336 participants (67.5%) showed an increased risk of chronic conditions with the WHR, among whom 105 were overweight (31.3%), and 123 had obesity (36.6%).

**TABLE 1:** Characteristics of the total sample and by BMI category at ages 41–48.

	Total sample (*n* = 499)	Under/normal weight^a^ (*n* = 204)	Overweight (*n* = 147)	Obesity (*n* = 147)	*p* ^b^
Age (years)	44.6 ± 1.6	44.8 ± 1.6	44.4 ± 1.6	44.5 ± 1.6	0.1
Average weekly physical activity levels					
Sedentary/low	174 (35.9)	53 (26.5)	58 (40.3)	63 (44.7)	0.007
Moderate	117 (24.1)	55 (27.5)	30 (20.8)	32 (22.7)	
Vigorous	194 (40)	92 (46)	56 (38.9)	46 (32.6)	
Presence of comorbidities					0.2
No	373 (74.9)	157 (77.3)	113 (76.9)	102 (69.4)	
Yes	125 (25.1)	46 (22.7)	34 (23.1)	45 (30.6)	
Respiratory conditions					0.07
No	295 (59.1)	130 (63.7)	89 (60.5)	76 (51.7)	
Yes	204 (80.9)	74 (36.3)	58 (39.5)	71 (48.3)	
Highest educational attainment					0.001
Certificate/diploma or less	126 (25.3)	38 (18.6)	31 (21.1)	56 (38.1)	
University degree	190 (38.1)	87 (42.7)	61 (41.5)	42 (28.6)	
Higher university degree	183 (36.7)	79 (38.7)	55 (37.4)	49 (33.3)	
Difficulty managing income					< 0.001
Impossible	152 (30.5)	46 (22.6)	37 (25.2)	68 (46.3)	
Not too bad	203 (40.7)	87 (42.7)	65 (44.2)	51 (34.7)	
Easy	144 (28.9)	71 (34.8)	45 (30.6)	28 (19.1)	
Marital status					0.02
Never married	58 (11.6)	17 (8.3)	12 (8.2)	28 (19.1)	
Married/de facto	390 (78.2)	164 (80.4)	120 (81.6)	106 (72.1)	
Separated/divorced/widowed	51 (10.2)	23 (11.3)	15 (10.2)	13 (8.8)	
Menopausal status					0.1
Pre‐menopausal	202 (40.6)	94 (46.1)	58 (39.7)	49 (33.3)	
Peri‐menopausal	99 (19.9)	38 (18.6)	27 (18.5)	34 (23.1)	
Post‐menopausal	13 (2.6)	2 (1.0)	7 (4.8)	4 (2.7)	
Unknown menopausal status	184 (37.0)	70 (34.3)	54 (37.0)	60 (40.8)	
Smoking status					0.004
Never smoker	344 (68.9)	152 (74.5)	105 (71.4)	86 (58.5)	
Ex/current smoker	155 (31.1)	52 (25.5)	42 (28.6)	61 (41.5)	
Alcohol drinking pattern					0.06
Never/rarely drinks	128 (25.7)	43 (21.1)	38 (25.9)	46 (31.3)	
Low‐risk drinker	343 (68.7)	154 (75.5)	99 (67.4)	90 (61.2)	
Risky/high risk drinker	28 (5.6)	7 (3.4)	10 (6.8)	11 (7.5)	
Height (cm)	165.4 ± 6.2	165.5 ± 6.4	165.7 ± 6.1	165.1 ± 6.1	0.7
Weight (cm)	76.1 ± 18.3	61.2 ± 6.1	75 ± 7	98 ± 15.8	< 0.001
Waist circumference (cm)	88.8 ± 14.8	76.6 ± 6.2	88.3 ± 6.6	106.3 ± 11.5	< 0.001
Hip circumference (cm)	106.2 ± 13.5	95.3 ± 6.3	106 ± 5.6	121.8 ± 11.1	< 0.001
Waist‐to‐hip ratio	0.83 ± 0.07	0.80 ± 0.06	0.83 ± 0.06	0.87 ± 0.07	< 0.001
BMI (kg/m^2^)	27.8 ± 6.4	22.3 ± 1.7	27.3 ± 1.3	35.9 ± 5.2	< 0.001
Annual percent change in weight (%/year)	0.8 ± 0.9	0.4 ± 0.6	0.7 ± 0.9	1.4 ± 1.0	< 0.001
Waist circumference risk					
Low risk (< 80 cm)	165 (33.1)	151 (74)	13 (8.8)	0 (0)	< 0.001
High risk (≥ 80 cm)	334 (66.9)	53 (26)	134 (91.2)	147 (100)	
Waist‐to‐hip ratio risk					
Low risk (< 0.8)	162 (32.5)	96 (47.1)	42 (28.6)	23 (15.8)	< 0.001
High risk (≥ 0.8)	336 (67.5)	108 (52.9)	105 (71.4)	123 (84.3)	
Worse handgrip strength performance	164 (33.7)	76 (38.2)	44 (31)	44 (30.6)	0.2
Worse chair rise performance	169 (34.1)	65 (31.9)	37 (25.3)	67 (46.5)	< 0.001
Worse standing balance performance	201 (40.4)	67 (32.8)	56 (38.1)	78 (53.4)	< 0.001
Physical function test results by performance group					
Handgrip strength (kg)					
Better performance	34.0 (32.0–36.0)	33.0 (30.0–36.0)	34.0 (32.0–36.0)	32.0 (31.0–36.0)	0.2
Worse performance	26.0 (24.0–28.0)	26.0 (24.0–28.0)	26.0 (24.0–28.0)	26.0 (23.0–28.0)	0.4
Chair rise reciprocal time (s^−1^)					
Better performance	7.1 (6.3–8.3)	7.1 (6.3–8.3)	7.1 (6.3–8.3)	7.1 (5.9–8.3)	0.6
Worse performance	4.8 (4.3–5.0)	4.8 (4.5–5.3)	4.8 (4.3–5.0)	4.5 (4.2–5.0)	0.02
Standing balance (s)					
Better performance	14.0 (7.0–30.0)	20.0 (9.0–30.0)	12.0 (7.0–27.0)	10.0 (6.0–14.5)	< 0.001
Worse performance	2.0 (2.0–3.0)	2.0 (2.0–3.0)	3.0 (2.0–4.0)	2.0 (2.0–3.0)	0.02

*Note:* Presence of comorbidities: self‐reported diagnosis or treatment for osteoarthritis, rheumatoid arthritis or other arthritis, diabetes, heart disease, and/or cancer. Respiratory conditions: self‐reported diagnosis or treatment for asthma or bronchitis. Values are presented as *n* (%) for categorical variables, mean ± SD for normally distributed continuous variables, and median (IQR) for non‐normally distributed continuous variables. Worse handgrip strength, chair rise and standing balance performances defined by the lowest tertile of the sample (vs. better performance, represented by the two highest tertiles).

Abbreviation: BMI, body mass index.

^a^ Only 2 participants were classified as underweight (BMI < 18.5 kg/m^2^).

^b^ *p*‐values calculated using chi‐square tests for categorical covariates, ANOVA for comparisons of means and Kruskal–Wallis tests for comparisons of medians across BMI groups.

The mean handgrip strength of the sample was 31.3 ± 5.7 kg, the chair rise reciprocal time was 6.5 ± 1.9 s, and the median standing balance time with eyes closed was 6 s (IQR: 3, 18). Participants were allocated to two groups based on the sample's tertiles, with the lowest tertile representing the ‘worse performance’ group, and the two highest tertiles representing the ‘better performance’ group. LOWESS scatterplots illustrating the relationships between anthropometric indicators and performance‐based physical function tests are shown in Appendix 2, Supplementary Figures 3–5. Overall, handgrip strength increased with height, while associations with weight and adiposity indicators (WC, WHR) were comparatively weak and largely flat. In contrast, chair‐rise and standing‐balance performances tended to decrease with increasing weight, BMI, WC, and WHR.

### 3.1 Cross‐Sectional Analyses

In the fully adjusted Poisson regression models, women with obesity at ages 41–48 had around 50% higher prevalence of worse chair rise and standing balance performances than those who were under/normal weight (Table 2). Compared with the low‐risk WC group, women in the high‐risk WC group had a 24% lower prevalence of worse handgrip strength, but a 41% and 43% higher prevalence of worse chair rise and standing balance performance, respectively. Those in the high‐risk WHR group had 1.54 times the prevalence of worse chair performance. No significant associations were observed between WHR and handgrip strength or standing balance, or between BMI category and handgrip strength.

**TABLE 2:** Prevalence ratios (PR) and 95% confidence intervals (95% CI) for worse performance^a^ on the handgrip strength, chair rise, and standing balance tests across anthropometric measurement categories at ages 41–48 (cross‐sectional analyses, *n* = 499).

Anthropometric measurement	Unadjusted	Model 1^b^	Model 2^c^
	PR (95% CI)	PR (95% CI)	PR (95% CI)
Handgrip strength			
BMI category			
Under/Normal weight	(Ref.)	(Ref.)	(Ref.)
Overweight	0.81 (0.60, 1.10)	0.88 (0.65, 1.19)	0.85 (0.63, 1.15)
Obesity	0.80 (0.59, 1.08)	0.80 (0.59, 1.09)	0.73 (0.53, 1.01)
WC risk			
Low‐risk WC (< 80 cm)	(Ref.)	(Ref.)	(Ref.)
High‐risk WC (≥ 80 cm)	0.76 (0.59, 0.98)*	0.80 (0.62, 1.04)	0.76 (0.59, 0.99)*
WHR risk			
Low‐risk WHR (< 0.8)	(Ref.)	(Ref.)	(Ref.)
High‐risk WHR (≥ 0.8)	0.80 (0.62, 1.02)	0.84 (0.64, 1.10)	0.82 (0.62, 1.07)
Chair rise			
BMI category			
Under/normal weight	(Ref.)	(Ref.)	(Ref.)
Overweight	0.80 (0.56, 1.12)	1.01 (0.75, 1.38)	1.03 (0.76, 1.41)
Obesity	1.46 (1.12, 1.91)**	1.68 (1.33, 2.11)***	1.55 (1.21, 1.99)***
WC risk			
Low‐risk WC (< 80 cm)	(Ref.)	(Ref.)	(Ref.)
High‐risk WC (≥ 80 cm)	1.47 (1.09, 1.96)*	1.49 (1.15, 1.91)**	1.41 (1.09, 1.83)**
WHR risk			
Low‐risk WHR (< 0.8)	(Ref.)	(Ref.)	(Ref.)
High‐risk WHR (≥ 0.8)	2.24 (1.58, 3.17)***	1.58 (1.11, 2.26)**	1.54 (1.06, 2.22)**
Standing balance			
BMI category			
Under/normal weight	(Ref.)	(Ref.)	(Ref.)
Overweight	1.16 (0.87, 1.54)	1.23 (0.93, 1.63)	1.21 (0.91, 1.61)
Obesity	1.63 (1.27, 2.08)***	1.62 (1.26, 2.08)***	1.53 (1.16, 2.02)**
WC risk			
Low‐risk WC (< 80 cm)	(Ref.)	(Ref.)	(Ref.)
High‐risk WC (≥ 80 cm)	1.58 (1.21, 2.06)**	1.50 (1.15, 1.95)**	1.43 (1.09, 1.87)**
WHR risk			
Low‐risk WHR (< 0.8)	(Ref.)	(Ref.)	(Ref.)
High‐risk WHR (≥ 0.8)	1.62 (1.23, 2.12)***	1.38 (1.03, 1.84)**	1.31 (0.98, 1.76)

Abbreviations: BMI, body mass index; WC, waist circumference; WHR, waist‐to‐hip ratio.

^a^ The worse performance group was defined as the lowest tertile of the sample.

^b^ Model 1: adjusted for clinic location, age and physical activity levels.

^c^ Model 2: Model 1 + presence of comorbidities, respiratory conditions, highest educational attainment, difficulty managing income, marital status, smoking status and alcohol drinking pattern.

* *p* < 0.05.

** *p* < 0.01.

*** *p* < 0.001.

### 3.2 Longitudinal Analyses

BMI trajectories from ages 18–23 to 40–45 are shown in Figure 1. Two trajectory groups were identified and labelled Normal‐Increasing (75.2%) and High‐Increasing (24.8%). Sample characteristics by BMI trajectory groups are shown in Appendix 2, Supplementary Table 3. The associations between BMI trajectory groups from ages 18–23 to 40–45 and performance at ages 41–48 are shown in Table 3. Compared with the Normal‐Increasing group, the High‐Increasing BMI group had a 42% higher prevalence of worse performance on both chair rise and standing balance tests. Associations with handgrip strength performance were non‐significant.

**FIGURE 1:**
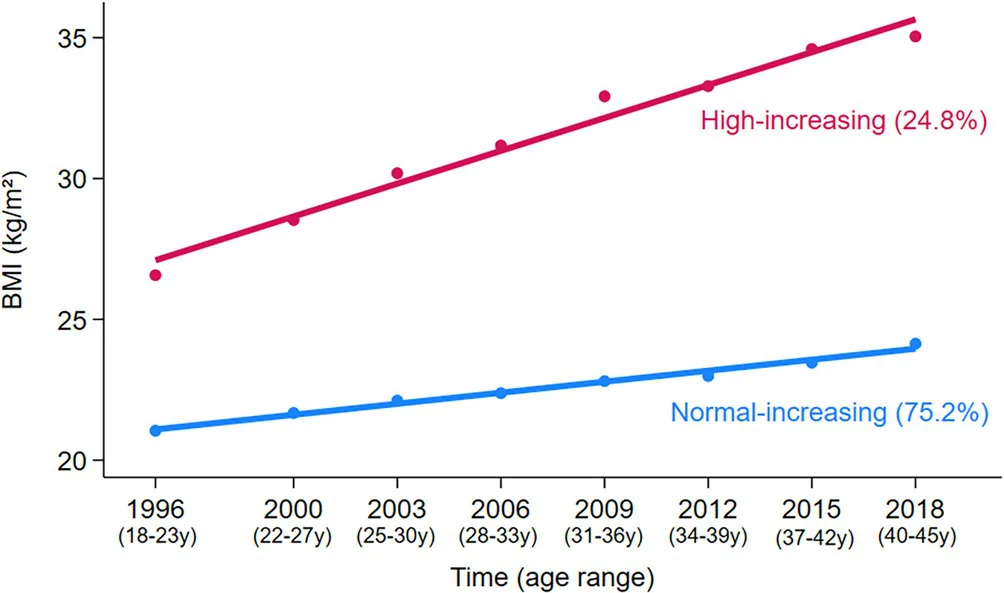
Results of the group‐based trajectory model, with two trajectories of body mass index progression over time. Solid lines represent model‐estimated BMI trajectories; dots represent observed mean BMI at each time point.

**TABLE 3:** Prevalence ratios (PR) and 95% confidence intervals (95% CI) for worse performance^a^ on the handgrip strength, chair rise, and standing balance tests by BMI trajectory groups (longitudinal analysis, *n* = 499).

Model/BMI trajectory group	Handgrip strength	Chair rise	Standing balance
	PR (95% CI)	PR (95% CI)	PR (95% CI)
Unadjusted			
Normal‐increasing	(Ref.)	(Ref.)	(Ref.)
High‐increasing	0.84 (0.61, 1.14)	1.49 (1.16, 1.91)*	1.49 (1.20, 1.84)**
Model 1^b^			
Normal‐increasing	(Ref.)	(Ref.)	(Ref.)
High‐increasing	0.87 (0.63, 1.20)	1.55 (1.21, 1.99)**	1.54 (1.22, 1.93)**
Model 2^c^			
Normal‐increasing	(Ref.)	(Ref.)	(Ref.)
High‐increasing	0.79 (0.56, 1.12)	1.42 (1.09, 1.84)*	1.42 (1.10, 1.84)*

Abbreviation: BMI, body mass index.

^a^ The worse performance group was defined as the lowest tertile of the sample.

^b^ Model 1: Adjusted by clinic location, age at baseline and final follow‐up, and physical activity levels at baseline and final follow‐up.

^c^ Model 2: Model 1 + presence of comorbidities, respiratory conditions, highest educational attainment, difficulty managing income, marital status, smoking status and alcohol drinking pattern at final follow‐up.

* *p* < 0.01.

** *p* < 0.001.

The average annual percent increase in weight of the whole sample was 0.77% per year (95% CI: 0.68, 0.85). The Normal‐Increasing BMI group had a mean percent increase of 0.62% per year (95% CI: 0.54, 0.70), while the High‐Increasing BMI group showed a 1.23% annual increase (95% CI: 1.00, 1.45). Each one‐percentage‐point increase in weight was associated with a 21% higher prevalence of worse standing balance performance in the final model (Table 4). There were no significant associations with handgrip strength or chair‐rise performance.

**TABLE 4:** Prevalence ratios (PR) and 95% confidence intervals (95% CI) for worse performance^a^ on the handgrip strength, chair rise, and standing balance tests per one‐percentage‐point increase in average annual weight change (longitudinal analysis, *n* = 491).

Model/annual weight change	Handgrip strength	Chair rise	Standing balance
	PR (95% CI)	PR (95% CI)	PR (95% CI)
Unadjusted	0.89 (0.78, 1.02)	1.17 (1.03, 1.33)*	1.08 (0.96, 1.23)
Model 1^b^	0.94 (0.80, 1.10)	1.10 (0.95, 1.28)	1.21 (1.05, 1.39)**
Model 2^c^	0.93 (0.79, 1.08)	1.09 (0.96, 1.25)	1.21 (1.06, 1.38)**

Abbreviation: BMI, body mass index.

^a^ The worse performance group was defined as the lowest tertile of the sample.

^b^ Model 1: adjusted by clinic location, age at baseline and final follow‐up, height, and physical activity levels at baseline and final follow‐up.

^c^ Model 2: Model 1 + presence of comorbidities, respiratory conditions, highest educational attainment, difficulty managing income, marital status, smoking status and alcohol drinking pattern at final follow‐up.

* *p* < 0.05.

** *p* < 0.01.

Additional adjustment for menopausal status in sensitivity analyses did not materially alter results, nor did alternative physical activity adjustment models or using imputed datasets (Appendix 2, Supplementary Tables 4–6).

## 4 Discussion

This study evaluated cross‐sectional associations between weight indicators and performance on physical function tests in midlife, as well as longitudinal associations between BMI trajectories, annual weight gain percentage from early to mid‐adulthood and performance in mid‐aged Australian women. BMI‐defined obesity in midlife was associated with worse performance on the chair rise and standing balance tests, as were high‐risk waist circumference and waist‐to‐hip ratio. Two BMI trajectories were identified over more than two decades of follow‐up: the Normal‐Increasing (~75%) and High‐Increasing (~25%) groups. Those in the second group also showed significantly worse performance on the chair rise and standing balance tests in their forties. No associations with handgrip strength were observed. Our findings indicate that overweight and obesity from early adulthood may signal increased risk of poor physical function later in life.

Obesity is a major public health challenge worldwide and is associated with increased muscle fat infiltration and increased risk of sarcopenia [30]. However, its effects on women's physical function throughout life are not fully understood. While higher BMI has been associated with better handgrip strength in midlife, obesity has also been linked to faster declines in strength and lower muscle quality in older age, determined as handgrip strength/arm lean mass [18, 31, 32, 33]. Sun et al. [9] highlighted the complex relationship between adiposity and frailty and suggested that body fat may be more significantly linked to a higher risk of frailty in women. Similarly, after finding that abdominal obesity was associated with accelerated muscle strength decline in men but not women from the English Longitudinal Study of Ageing, de Carvalho et al. [34] suggested WHR may be particularly relevant to women's muscle function, given sex differences in fat distribution. In the present study, both greater weight and central adiposity, as measured by WC and WHR, were cross‐sectionally associated with worse performance in the chair rise and standing balance tests.

Previous data from ALSWH show a continuing trend of increasing rate of weight gain across cohorts that has profound consequences for women's health [35, 36]. Midlife is considered a particularly high‐risk time for weight gain, which may be exacerbated by factors such as energy imbalance, considering physical activity, sitting time and energy intake, and the menopausal transition [36, 37]. Nevertheless, biological and behavioural determinants of weight gain also relate to psychological, social, environmental and economic factors [6, 36]. In the present study, women in the High‐Increasing BMI trajectory showed lower education, more difficulties managing income and a higher proportion of never married than those with Normal‐Increasing BMI. As argued by Darling et al. [38], weight trajectories are set early in life, and effective preventive strategies must work on many levels, but particularly in early life's social determinants and the obesogenic environment.

### 4.1 Strengths and Limitations

One of the main strengths of this study is the inclusion of three performance‐based physical function tests that can independently predict disability in older age [39]. M‐PreM also included central obesity measures, and repeated height and weight measurements across adulthood in ALSWH. Sample‐derived tertiles were used to identify relatively poorer performance while maintaining adequate group sizes in this relatively small, young, and high‐functioning cohort, for whom clinically meaningful cut‐points are limited, particularly for chair rise and standing balance [40]. However, this approach may limit clinical interpretability and comparability with studies using established cut‐points. Generalisability may also be limited, as COVID‐19 restrictions meant that clinical assessments were performed in less than 40% of the final M‐PreM sample. Thus, trajectory group distributions may not reflect that of the broader ALSWH 1973–1978 cohort. The low prevalence of individual conditions required comorbidity to be represented as a binary composite, potentially resulting in residual confounding and/or overadjustment. Finally, associations with underweight could not be assessed because only two women met this criterion.

## 5 Conclusions

In summary, high‐increasing BMI trajectories and greater annual weight gain from early to mid‐adulthood were significantly associated with worse performance on two of three physical function tests among Australian mid‐aged women, namely the chair rise and standing balance tests. Cross‐sectional measures of obesity and central adiposity were also associated with worse performance on both tests at age 41–48. These findings suggest that long‐term patterns of weight gain may be markers of poorer midlife physical performance, although causal interpretations and implications for subsequent functional decline require further investigation.

## Author Contributions

L.W.R. was responsible for the analysis and preparation of this manuscript. G.D.M., J.D. and G.I.M. provided supervision and support in the study conception, design, and interpretation of results. All authors reviewed the results and approved the final version of the manuscript.

## Funding

This work was supported by the National Health and Medical Research Council [grant numbers APP1000986, APP1129592, APP2009577, APP2008702, and APP1153420]. The funding sources were not involved in the research and preparation of the article.

## Conflicts of Interest

The authors declare no conflicts of interest.

## Supporting information


**Text S1:** Appendix 1: Additional details on study participation.
**Supplementary Figure1**. Participant flowchart.
**Supplementary Table 1**. Characteristics of the M‐PreM participants and the remainder of the ALSWH 1973–1978 cohort in the eighth ALSWH survey (2018, ages 40–45).
**Text S2:** Appendix 2: Additional details on study measures.
**Supplementary Figure 2**. Directed cyclic graph (DAG) with feedback loops, illustrating the relationships between BMI (exposure), physical performance (outcome), and the covariates.
**Supplementary Table 2**. Sample characteristics between those with complete and incomplete covariates data.
**Supplementary Table 3**. Sample characteristics by BMI trajectory group.
**Supplementary Figure 3**. LOWESS scatterplots showing the cross‐sectional associations between (A) height (cm), (B) weight (kg), (C) body mass index (BMI, kg/m^2^), (D) waist circumference (cm), and (E) waist‐to‐hip ratio (WHR) and performance on the handgrip strength test at age 41–48.
**Supplementary Figure 4**. LOWESS scatterplots showing the cross‐sectional associations between (A) height (cm), (B) weight (kg), (C) body mass index (BMI, kg/m^2^), (D) waist circumference (cm), and (E) waist‐to‐hip ratio (WHR) and performance on the chair rise test (reciprocal time, sec^−1^) at age 41–48.
**Supplementary Figure 5**. LOWESS scatterplots showing the cross‐sectional associations between (A) height (cm), (B) weight (kg), (C) body mass index (BMI, kg/m^2^), (D) waist circumference (cm), and (E) waist‐to‐hip ratio (WHR) and performance on the standing balance test with eyes closed (sec) at age 41–48.
**Supplementary Table 4**. Sensitivity analysis for the associations between BMI trajectories and percent annual change in weight with worse performance^a^ on physical function tests, including additional adjustment for menopausal status.
**Supplementary Table 5**. Sensitivity analysis using alternative physical activity (PA) adjustment models for the associations between BMI trajectories and worse performance on physical function tests.
**Supplementary Table 6**. Sensitivity analysis using complete‐case and multiply imputed datasets for the associations between BMI trajectories and percent annual change in weight with worse performance on physical function tests.

## Data Availability

ALSWH survey data are owned by the Australian Government Department of Health, Disability and Ageing and due to the personal nature of the data collected, release by ALSWH is subject to strict contractual and ethical restrictions. Ethical review of ALSWH is by the Human Research Ethics Committees at The University of Queensland and The University of Newcastle. De‐identified data are available to collaborating researchers where a formal request to make use of the material has been approved by the ALSWH Data Access Committee. The committee is receptive of requests for datasets required to replicate results. Information on applying for ALSWH data is available from https://alswh.org.au/for‐data‐users/applying‐for‐data/.
