# Supplementary material for: Obesity and Midlife Performance: A Cross‐Sectional and Longitudinal Analysis of Women in the Menarche‐To‐PreMenopause Study
Source: Diabetes Obes Metab. 2026 Sep 14;28(12):11373–83. doi: 10.1111/dom.71349 (PMC13730985; doi:10.1111/dom.71349)
Supplement: Supplementary file 1 [file DOM-28-11373-s001.docx]

**Supplementary Data**

**Contents**

**Appendix 1**

- Additional details on study participation.
- Supplementary Figure 1. Participant flowchart.
- Supplementary Table 1. Characteristics of the M-PreM participants and the remainder of the ALSWH 1973–1978 cohort in the eighth ALSWH survey

**Appendix 2**

- Additional details on study measures.
- Supplementary Figure 2. Directed cyclic graph (DAG) with feedback loops, illustrating the relationships between the primary exposure, the outcome, and the covariates.
- Supplementary Table 2. Sample characteristics between those with complete and incomplete covariates data.
- Supplementary Table 3. Sample characteristics by BMI trajectory group.
- Supplementary Figure 3. LOWESS scatterplots of the relationship between (A) height (cm), (B) weight (kg), (C) body mass index (BMI, kg/m2), (D) waist circumference (cm), and (E) waist-to-hip ratio (WHR) with performance on the handgrip strength test.
- Supplementary Figure 4. LOWESS scatterplots of the relationship between (A) height (cm), (B) weight (kg), (C) body mass index (BMI, kg/m2), (D) waist circumference (cm), and (E) waist-to-hip ratio (WHR) with performance on the chair rise test (reciprocal time, 1/sec).
- Supplementary Figure 5. LOWESS scatterplots of the relationship between (A) height (cm), (B) weight (kg), (C) body mass index (BMI, kg/m2), (D) waist circumference (cm), and (E) waist-to-hip ratio (WHR) with performance on the standing balance test.
- Supplementary Table 4. Sensitivity analysis for the associations between BMI trajectories and percent annual change in weight with worse performance on physical function tests, including additional adjustment for menopausal status.
- Supplementary Table 5. Sensitivity analysis using alternative physical activity (PA) adjustment models for the associations between BMI trajectories and percent annual change in weight with worse performance on physical function tests.
- Supplementary Table 6. Sensitivity analysis using complete-case and multiply imputed datasets for the associations between BMI trajectories and percent annual change in weight with worse performance on physical function tests.

**Appendix 1**

***Additional details on study participation***

**Supplementary Figure 1.** Participant flowchart.

**Supplementary Table 1.** Characteristics of the M-PreM participants and the remainder of the ALSWH 1973–1978 cohort in the eighth ALSWH survey (2018, ages 40-45).

| **Variables** | **M-PreM clinical assessment**  **(n= 499)** | **M-PreM self-assessment sample**  **(n=779)** | **ALSWH 1973-78 cohort (n=5,843)** | **p-value*** |
| --- | --- | --- | --- | --- |
| Age (years) | 42.7 ± 1.5 | 42.7 ± 1.5 | 42.9 ± 1.5 | 0.95 |
| Missing | 0 | 0 | 0 |  |
| Height (cm) | 165.6 ± 6.2 | 166.2 ± 7.0 | 165.8 ± 7.1 | 0.29 |
| Missing | 0 | 2 | 69 |  |
| Weight (kg) | 73.7 ± 17.9 | 75.1 ± 17.7 | 76.8 ± 19.5 | <0.001 |
| Missing | 3 | 6 | 221 |  |
| BMI (kg/m²) | 26.8 ± 6.3 | 27.2 ± 6.3 | 27.9 ± 6.9 | <0.001 |
| Missing | 3 | 8 | 286 |  |
| Aria Plus grouped |  |  |  | <0.001 |
| Major cities | 463 (93.2) | 588 (76.0) | 3248 (57.4) |  |
| Inner / Outer regional | 30 (6.0) | 180 (23.3) | 2289 (40.5) |  |
| Remote / Very remote | 4 (0.8) | 6 (0.8) | 122 (2.2) |  |
| Missing | 2 | 5 | 184 |  |
| Highest educational attainment |  |  |  | <0.001 |
| Certificate/Diploma or less | 126 (25.3) | 336 (43.1) | 2420 (44.0) |  |
| University degree | 190 (38.1) | 254 (32.6) | 1713 (31.2) |  |
| Higher university degree | 183 (36.7) | 189 (24.3) | 1365 (24.8) |  |
| Missing | 0 | 0 | 345 |  |
| Difficulty managing income |  |  |  | <0.001 |
| Impossible/difficult | 152 (30.5) | 305 (39.2) | 2230 (40.6) |  |
| Not too bad | 203 (40.7) | 298 (38.3) | 2171 (39.5) |  |
| Easy | 144 (28.9) | 175 (22.5) | 1092 (19.9) |  |
| Missing | 0 | 1 | 350 |  |
| Marital status |  |  |  | 0.71 |
| Never married | 58 (11.6) | 77 (9.9) | 636 (11.6) |  |
| Married / De facto | 390 (78.2) | 624 (80.1) | 4299 (78.1) |  |
| Separated / Divorced / Widowed | 51 (10.2) | 78 (10.0) | 571 (10.4) |  |
| Missing | 0 | 0 | 337 |  |
| Average weekly physical activity levels |  |  |  | 0.002 |
| Inactive | 174 (35.9) | 323 (43.8) | 2231 (44.1) |  |
| Active | 311 (64.1) | 414 (56.2) | 2824 (55.9) |  |
| Missing | 14 | 42 | 788 |  |
| Smoking status |  |  |  | <0.001 |
| Never smoker | 344 (68.9) | 482 (62.0) | 3428 (61.0) |  |
| Ex-smoker | 125 (25.1) | 235 (30.2) | 1587 (28.2) |  |
| Current smoker | 30 (6.0) | 61 (7.8) | 605 (10.8) |  |
| Missing | 0 | 1 | 223 |  |
| Alcohol drinking pattern |  |  |  | <0.001 |
| Non-drinker/Rarely drinks | 128 (25.7) | 259 (33.3) | 1966 (34.9) |  |
| Low risk drinker | 343 (68.7) | 468 (60.2) | 3241 (57.6) |  |
| Risky / High risk drinker | 28 (5.6) | 51 (6.6) | 419 (7.5) |  |
| Missing | 0 | 1 | 217 |  |
| Presence of comorbidities |  |  |  | 0.03 |
| No | 342 (69.4) | 494 (64.3) | 3571 (63.3) |  |
| Yes | 151 (30.6) | 274 (35.7) | 2070 (36.7) |  |
| Missing | 7 | 13 | 265 |  |

Values are presented as n (%) for categorical variables and mean ± SD for normally distributed continuous variables. ALSWH: Australian Longitudinal Study on Women’s Health. BMI: body mass index. M-PreM: Menarche-to-PreMenopause Study. Comorbidities defined as self-reported diagnosis or treatment for one or more of the following: osteoarthritis, rheumatoid arthritis or other arthritis, diabetes, heart disease, asthma, bronchitis, and/or cancer. *P-values from one-way ANOVA and chi-square tests.

**Appendix 2**

***Additional details on study measures***

**Supplementary Figure 2.** Directed cyclic graph (DAG) with feedback loops, illustrating the relationships between BMI (exposure), physical performance (outcome), and the covariates.

BMI: body mass index. Bidirectional arrows highlight the reciprocal association and potential reverse causality between the exposure and certain confounding factors. Developed on daggity: https://www.dagitty.net/

**Supplementary Table 2.** Sample characteristics between those with complete and incomplete covariates data.

| **Variables** | **Complete (n=404)** | **Incomplete (n=95)** | **p-value** |
| --- | --- | --- | --- |
| Age (years) | 44.7 ± 1.6 | 44.2 ± 1.6 | 0.01 |
| Average weekly physical activity levels | | | 0.28 |
| Sedentary/Low | 143 (35.4) | 31 (38.3) |  |
| Moderate | 103 (25.5) | 14 (17.3) |  |
| Vigorous | 158 (39.1) | 36 (44.4) |  |
| Presence of comorbidities | | | 0.67 |
| No | 301 (74.5) | 72 (76.6) |  |
| Yes | 103 (25.5) | 22 (23.4) |  |
| Respiratory conditions | | | 0.85 |
| No | 238 (58.9) | 57 (60.0) |  |
| Yes | 166 (41.1) | 38 (40.0) |  |
| Highest educational attainment | | | 0.25 |
| Certificate/Diploma or less | 96 (23.8) | 30 (31.6) |  |
| University degree | 155 (38.4) | 35 (36.8) |  |
| Higher university degree | 153 (37.9) | 30 (31.6) |  |
| Difficulty managing income | | | 0.02 |
| Impossible | 112 (27.7) | 40 (42.1) |  |
| Not too bad | 172 (42.6) | 31 (32.6) |  |
| Easy | 120 (29.7) | 24 (25.3) |  |
| Marital status | |  | 0.82 |
| Never married | 46 (11.4) | 12 (12.6) |  |
| Married/De facto | 318 (78.7) | 72 (75.8) |  |
| Separated/Divorced/Widowed | 40 (9.9) | 11 (11.6) |  |
| Smoking status | |  | 0.01 |
| Never smoker | 290 (71.8) | 54 (56.8) |  |
| Ex/Current smoker | 114 (28.2) | 41 (43.2) |  |
| Alcohol drinking pattern | | | 0.52 |
| Never/Rarely drinks | 107 (26.5) | 21 (22.1) |  |
| Low-risk drinker | 277 (68.6) | 66 (69.5) |  |
| Risky/High risk drinker | 20 (5.0) | 8 (8.4) |  |
| Height (cm) | 165.4 ± 6.2 | 165.6 ± 6.2 | 0.84 |
| Weight (cm) | 76.4 ± 18.6 | 75.1 ± 17.2 | 0.55 |
| Waist circumference (cm) | 89.0 ± 15.0 | 88.0 ± 14.1 | 0.57 |
| Hip circumference (cm) | 106.7 ± 13.7 | 104.2 ± 12.3 | 0.11 |
| Waist-to-hip ratio | 0.83 ± 0.07 | 0.84 ± 0.07 | 0.34 |
| BMI (kg/m2) | 27.9 ± 6.5 | 27.4 ± 5.9 | 0.46 |
| Annual percent change in weight (%/year) | 0.8 ± 0.8 | 0.6 ± 1.3 | 0.07 |
| Waist circumference risk | | | 0.41 |
| Low risk (<80 cm) | 137 (33.9) | 28 (29.5) |  |
| High risk (≥ 80 cm) | 267 (66.1) | 67 (70.5) |  |
| Waist-to-hip ratio risk | |  | 0.17 |
| Low risk (<0.8) | 137 (33.9) | 25 (26.6) |  |
| High risk (≥ 0.8) | 267 (66.1) | 69 (73.4) |  |
| Worse handgrip strength performance | 132 (33.4) | 32 (35.2) | 0.75 |
| Worse chair rise performance | 135 (33.8) | 34 (35.8) | 0.71 |
| Worse standing balance performance | 160 (39.7) | 41 (43.2) | 0.54 |

BMI: body mass index. Presence of comorbidities: self-reported diagnosis or treatment for osteoarthritis, rheumatoid arthritis or other arthritis, diabetes, heart disease, and/or cancer. Respiratory conditions: self-reported diagnosis or treatment for asthma or bronchitis. Values are presented as n (%) for categorical variables, mean ± SD for normally distributed continuous variables, and median (IQR) for non-normally distributed continuous variables. **p-values calculated using chi-square tests for categorical covariates and t-tests for comparisons of means across two groups.

**Supplementary Table 3.** Sample characteristics by BMI trajectory group.

| **Variables** | **Normal-Stable BMI (n=377)** | **High-Increasing BMI (n=122)** | **p-value** |
| --- | --- | --- | --- |
| Age (years) | 44.6 ± 1.60 | 44.5 ± 1.65 | 0.28 |
| Average weekly physical activity levels |  |  | 0.06 |
| Sedentary/Low | 121 (33.0) | 53 (44.9) |  |
| Moderate | 92 (25.1) | 25 (21.2) |  |
| Vigorous | 154 (42.0) | 40 (33.9) |  |
| Presence of comorbidities |  |  | 0.13 |
| No | 288 (76.6) | 85 (69.7) |  |
| Yes | 88 (23.4) | 37 (30.3) |  |
| Respiratory conditions |  |  | 0.02 |
| No | 234 (62.1) | 61 (50) |  |
| Yes | 143 (37.9) | 61 (50) |  |
| Highest educational attainment |  |  | <0.001 |
| Certificate/Diploma or less | 77 (20.4) | 49 (40.2) |  |
| University degree | 151 (40.1) | 39 (32.0) |  |
| Higher university degree | 149 (39.5) | 34 (27.9) |  |
| Difficulty managing income |  |  | <0.001 |
| Impossible | 100 (26.5) | 52 (42.6) |  |
| Not too bad | 154 (40.9) | 49 (40.2) |  |
| Easy | 123 (32.6) | 21 (17.2) |  |
| Menopausal status |  |  | 0.69 |
| Pre-menopausal | 158 (42.0) | 44 (36.1) |  |
| Peri-menopausal | 72 (19.2) | 27 (22.1) |  |
| Post-menopausal | 10 (2.7) | 3 (2.5) |  |
| Unknown menopausal status | 136 (36.2) | 48 (39.3) |  |
| Marital status |  |  | 0.002 |
| Never married | 33 (8.8) | 25 (20.5) |  |
| Married/De facto | 304 (80.6) | 86 (70.5) |  |
| Separated/Divorced/Widowed | 40 (10.6) | 11 (9.0) |  |
| Smoking status |  |  | 0.02 |
| Never smoker | 270 (71.6) | 74 (60.7) |  |
| Ex/Current smoker | 107 (28.4) | 48 (39.3) |  |
| Alcohol drinking pattern |  |  | 0.04 |
| Never/Rarely drinks | 85 (22.6) | 43 (35.3) |  |
| Low-risk drinker | 270 (71.6) | 73 (59.8) |  |
| Risky/High risk drinker | 22 (5.8) | 6 (4.9) |  |
| Height (cm) | 165.7 ± 6.2 | 164.7 ± 6.1 | 0.15 |
| Weight (cm) | 68.8 ± 11.2 | 98.7 ± 17.4 | <0.001 |
| Waist circumference (cm) | 82.9 ± 9.9 | 106.8 ± 12.9 | <0.001 |
| Hip circumference (cm) | 101.1 ± 9.2 | 122.1 ± 12.3 | <0.001 |
| Waist-to-hip ratio | 0.82 ± 0.06 | 0.88 ± 0.07 | <0.001 |
| BMI (kg/m^2^) | 25.0 ± 3.6 | 36.3 ± 5.7 | <0.001 |
| Annual percent change in weight (%/year) | 0.6 ± 0.04 | 1.2 ± 0.1 | <0.001 |
| Waist circumference risk, n (%) |  |  | <0.001 |
| Low risk (<80 cm) | 164 (43.5) | 1 (0.8) |  |
| High risk (≥ 80 cm) | 213 (56.5) | 121 (99.2) |  |
| Waist-to-hip ratio risk, n (%) |  |  | <0.001 |
| Low risk (<0.8) | 141 (37.4) | 21 (17.4) |  |
| High risk (≥ 0.8) | 236 (62.6) | 100 (82.6) |  |
| Handgrip strength (kg) | 31 (28-35) | 32 (28-35) | 0.86 |
| Chair rise reciprocal time (sec) | 6.3 (5.3-8.3) | 5.6 (4.8-7.1) | <0.001 |
| Standing balance with eyes closed (sec) | 7 (3-22) | 4 (2-8) | <0.001 |

BMI: body mass index. Presence of comorbidities: self-reported diagnosis or treatment for osteoarthritis, rheumatoid arthritis or other arthritis, diabetes, heart disease, and/or cancer. Respiratory conditions: self-reported diagnosis or treatment for asthma or bronchitis. Values are presented as n (%) for categorical variables, mean ± SD for normally distributed continuous variables, and median (IQR) for non-normally distributed continuous variables. **p-values calculated using chi-square tests for categorical covariates, t-tests for comparisons of means and Kruskal-Wallis tests for comparisons of medians across BMI trajectory groups.

**Supplementary Figure 3.** LOWESS scatterplots showing the cross-sectional associations between (A) height (cm), (B) weight (kg), (C) body mass index (BMI, kg/m^2^), (D) waist circumference (cm), and (E) waist-to-hip ratio (WHR) and performance on the handgrip strength test at age 41-48.

Note: Dots represent observed values, and red lines represent LOWESS-smoothed curves.

**Supplementary Figure 4.** LOWESS scatterplots showing the cross-sectional associations between (A) height (cm), (B) weight (kg), (C) body mass index (BMI, kg/m^2^), (D) waist circumference (cm), and (E) waist-to-hip ratio (WHR) and performance on the chair rise test (reciprocal time, sec^-1^) at age 41-48.

Note: Dots represent observed values, and red lines represent LOWESS-smoothed curves.

**Supplementary Figure 5.** LOWESS scatterplots showing the cross-sectional associations between (A) height (cm), (B) weight (kg), (C) body mass index (BMI, kg/m^2^), (D) waist circumference (cm), and (E) waist-to-hip ratio (WHR) and performance on the standing balance test with eyes closed (sec) at age 41-48.

Note: Dots represent observed values, and red lines represent LOWESS-smoothed curves.

**Supplementary Table 4.** Sensitivity analysis for the associations between BMI trajectories and percent annual change in weight with worse performance^a^ on physical function tests, including additional adjustment for menopausal status.

|  | **Worse handgrip strength** | **Worse chair rise** | **Worse standing balance** |
| --- | --- | --- | --- |
| **Exposure variables** | **PR (95% CI)** | **PR (95% CI)** | **PR (95% CI)** |
| *Cross-sectional analyses* |  |  |  |
| **BMI category** |  |  |  |
| Under/normal weight | (ref.) | (ref.) | (ref.) |
| Overweight | 0.85 (0.62, 1.15) | 1.00 (0.74, 1.36) | 1.20 (0.90, 1.60) |
| Obesity | 0.73 (0.53, 1.01) | 1.53 (1.19, 1.96)** | 1.53 (1.16, 2.02)** |
| **WC risk** |  |  |  |
| Low-risk WC (<80 cm) | (ref.) | (ref.) | (ref.) |
| High-risk WC (≥80 cm) | 0.76 (0.58, 0.99)* | 1.39 (1.07, 1.80)* | 1.42 (1.08, 1.85)* |
| **WHR risk** |  |  |  |
| Low-risk WHR (<0.8) | (ref.) | (ref.) | (ref.) |
| High-risk WHR (≥0.8) | 0.81 (0.62, 1.06) | 1.52 (1.05, 2.20)* | 1.30 (0.97, 1.74) |
| *Longitudinal analyses* |  |  |  |
| BMI trajectory group |  |  |  |
| Normal-Increasing | (ref.) | (ref.) | (ref.) |
| High-Increasing | 0.80 (0.57, 1.12) | 1.44 (1.10, 1.87)** | 1.44 (1.12, 1.85)** |
| Average annual weight change | 0.92 (0.79, 1.08) | 1.10 (0.96, 1.26) | 1.22 (1.07, 1.39)** |

BMI: Body Mass Index. ^a^The worse performance group was defined as the lowest tertile of the sample. Models adjusted for clinic location, age (at baseline and final follow-up in longitudinal analyses), physical activity levels (at baseline and final follow-up in longitudinal analyses), presence of comorbidities, respiratory conditions, highest educational attainment, difficulty managing income, marital status, smoking status and alcohol drinking pattern at final follow-up. *p<0.05. **p<0.01.

**Supplementary Table 5.** Sensitivity analysis using alternative physical activity (PA) adjustment models for the associations between BMI trajectories and worse performance on physical function tests.

| **PA adjustment** | **PA at S2+S8** | **PA at S2 only** | **PA at S8 only** |
| --- | --- | --- | --- |
| **Exposure variables** | **PR (95% CI)** | **PR (95% CI)** | **PR (95% CI)** |
| BMI trajectory group |  |  |  |
| Worse handgrip strength | 0.79 (0.56, 1.12) | 0.85 (0.60, 1.19) | 0.74 (0.54, 1.03) |
| Worse chair rise | 1.42 (1.09, 1.84)** | 1.37 (1.06, 1.78)** | 1.47 (1.15, 1.86)** |
| Worse standing balance | 1.42 (1.10, 1.84)** | 1.55 (1.21, 1.98)** | 1.28 (1.01, 1.63)* |
| Average annual weight change |  |  |  |
| Worse handgrip strength | 0.93 (0.79, 1.08) | 0.95 (0.81, 1.11) | 0.85 (0.74, 0.97)* |
| Worse chair rise | 1.09 (0.96, 1.25) | 1.11 (0.97, 1.27) | 1.10 (0.98, 1.25) |
| Worse standing balance | 1.21 (1.06, 1.38)** | 1.22 (1.07, 1.39)** | 1.08 (0.96, 1.22) |

PA: physical activity. PR: prevalence ratio. 95% CI: 95% confidence interval. S2: Survey 2 (baseline PA). S8: Survey 8 (final follow-up PA). Models also adjusted for clinic location, age at baseline, age at clinical assessment, presence of comorbidities, respiratory conditions, highest educational attainment, difficulty managing income, marital status, smoking status and alcohol drinking pattern.*p<0.05. **p<0.01.

**Supplementary Table 6.** Sensitivity analysis using complete-case and multiply imputed datasets for the associations between between BMI trajectories and percent annual change in weight with worse performance on physical function tests.

|  | **Worse handgrip strength** | **Worse chair rise** | **Worse standing balance** |
| --- | --- | --- | --- |
| **Exposure variables** | **PR (95% CI)** | **PR (95% CI)** | **PR (95% CI)** |
| *Cross-sectional analyses* |  |  |  |
| **BMI category** |  |  |  |
| Under/normal weight | (ref.) | (ref.) | (ref.) |
| Overweight | 0.84 (0.62, 1.13) | 1.02 (0.76, 1.38) | 1.18 (0.89, 1.57) |
| Obesity | 0.73 (0.53, 1.02) | 1.49 (1.16, 1.90)** | 1.54 (1.18, 2.01)** |
| **WC risk** |  |  |  |
| Low-risk WC (<80 cm) | (ref.) | (ref.) | (ref.) |
| High-risk WC (≥80 cm) | 0.75 (0.58, 0.97)* | 1.42 (1.09, 1.84)** | 1.45 (1.11, 1.89)** |
| **WHR risk** |  |  |  |
| Low-risk WHR (<0.8) | (ref.) | (ref.) | (ref.) |
| High-risk WHR (≥0.8) | 0.81 (0.62, 1.06) | 1.51 (1.05, 2.17)* | 1.35 (1.01, 1.81)* |
| *Longitudinal analyses* |  |  |  |
| BMI trajectory group |  |  |  |
| Normal-Increasing | (ref.) | (ref.) | (ref.) |
| High-Increasing | 0.79 (0.58, 1.09) | 1.39 (1.10, 1.77)** | 1.35 (1.07, 1.69)* |
| Average annual weight change | 0.86 (0.76, 0.99)* | 1.11 (0.99, 1.25) | 1.07 (0.96, 1.20) |

BMI: Body Mass Index. ^a^The worse performance group was defined as the lowest tertile of the sample. Models adjusted for clinic location, age (at baseline and final follow-up in longitudinal analyses), physical activity levels (at baseline and final follow-up in longitudinal analyses), presence of comorbidities, respiratory conditions, highest educational attainment, difficulty managing income, marital status, smoking status and alcohol drinking pattern at final follow-up. *p<0.05. **p<0.01.
